# Meaningful organizational routines in primary healthcare: influencing factors and their consequences for routine enactment

**DOI:** 10.1108/JHOM-10-2023-0317

**Published:** 2024-12-19

**Authors:** Mattias Jacobsson, Malin Näsholm

**Affiliations:** Umeå School of Business, Economics and Statistics, Umeå University, Umeå, Sweden

**Keywords:** Organizational routines, Primary healthcare, Meaningfulness, Practice

## Abstract

**Purpose:**

Based on the well-known risks associated with deviating from established routines in primary healthcare and the positive consequences of upholding them, the purpose of this study is to increase the understanding of the role of meaningfulness in the enactment of organizational routines.

**Design/methodology/approach:**

The study is based on 24 semi-structured interviews with three different professional categories in primary healthcare in Sweden. The data were analyzed using thematic analysis on a latent level, combined with a two-factor model as sensitizing concepts.

**Findings:**

Differences are identified between factors that reduce meaninglessness (called “sufficiency factors”) and those that enable meaningfulness (called “meaningfulness factors”). Nine sufficiency factors and six meaningfulness factors explain what makes organizational routines perceived as meaningful by the different professional groups. A two-factor matrix is developed that highlights the intricate challenges associated with routine enactment based on these factors.

**Originality/value:**

The study is unique in that it is the first to integrate research on organizational routines and meaningfulness. However, understanding meaningful organizational routines is not only essential because it is an overlooked area in both of these two streams of research but also because of its clear, practical relevance in the primary healthcare setting.

## 1. Introduction

This paper focuses on “meaningful organizational routines” in primary healthcare. This can be defined as organizational routines that both enable work to be successfully executed (Feldman and Pentland, 2003) and provide organization members with a sense of meaningfulness in their daily practice (Bailey *et al.*, 2019). Delving into the meaningfulness of organizational routines is essential not only because it is an overlooked area of research in both routines and meaningfulness research but also because of routines’ distinct practical relevance (Greenhalgh, 2008) and the benefits associated with meaningfulness (Handoyo *et al.*, 2023) in the healthcare setting.

On the one hand, in research on meaningfulness, it is well established that there are significant benefits associated with meaningfulness in work, both for organizations in assuring performance and for the individual’s work engagement (Michaelson *et al.*, 2014). Meaningfulness in work, which can be defined as “… *the subjective sense of meaningfulness or meaninglessness that individuals derive from their work”* (Martikainen *et al.*, 2022, p. 1,138), has, for example, been found to improve commitment and motivation and reduce adverse outcomes such as intentions to resign or be absent from work (Steger *et al.*, 2012). Experiencing work as meaningful has also been found to give people the resources to better cope with stress (Allan *et al.*, 2016) and thus contribute to resilience (Handoyo *et al.*, 2023). Over the years, research on meaningful work has identified a large variety of factors functioning as prerequisites of meaningfulness (Bailey *et al.*, 2019; Lysova *et al.*, 2019). These factors relate to everything from organizational culture and leadership to the social context at work (Lysova *et al.*, 2019). Still, meaningfulness in work is elusive since it is inherently subjective (Bailey *et al.*, 2019) and because some factors are shown to promote meaningfulness, while others merely reduce non-meaningfulness (Bailey and Madden, 2016).

On the other hand, despite often being associated with something negative, like being monotonous or dull (Rytterstöm *et al.*, 2011; Greenhalgh, 2008), it is well known that organizational routines play a crucial role in upholding stability and continuity at work (Feldman and Pentland, 2003). In primary healthcare organizations, the empirical context of this paper, where the influx of patients with various illnesses is extensive and the staff turnover is often high, well-functioning organizational routines are even more crucial (Greenhalgh, 2008). For example, organizational routines are identified as fundamental for both patient safety and continuity of care in that they function as a “structuring device” for work activities and roles (Greenhalgh, 2008; Rytterstöm *et al.*, 2011; Rosales, 2020).

However, if organizational routines are not perceived as relevant or if staff feels unmotivated, there is a risk that they will deviate from or ignore them (Stiles *et al.*, 2015; Rosales, 2020), which could have hazardous consequences for both patients and staff (Banja, 2010). Despite the apparent risks associated with deviations in healthcare delivery (Banja, 2010) and the positive consequences of upholding routines (Greenhalgh, 2008), research on organizational routines and meaningful work has rarely been integrated, and knowledge regarding *how* individuals find meaning in performing organizational routines is lacking today. It is this practical challenge and subsequent theoretical research gap that motivates this research. Based on a qualitative study in Swedish primary healthcare, we aim *to increase the understanding of the role of meaningfulness in the enactment of organizational routines* by asking: *What characterizes organizational routines that are perceived as meaningful, and how does the perception of meaningfulness influence routine enactment?* In other words, the study engages in understanding what is needed for organizational routines to be perceived as meaningful and worth upholding despite their negative associations.

The empirical study consisted of semi-structured interviews with three professional categories in primary healthcare: managers/administrative staff, nurses/other medical staff and physicians. Based on a thematic analysis on a latent level, in combination with the notion of a two-factor model inspired by the findings of Bailey and Madden (2016), a differentiation is made between factors that reduce *meaninglessness* (called “sufficiency factors”) and factors that facilitate *meaningfulness* (called “meaningfulness factors”) in organizational routines. Fifteen factors are identified in total, nine sufficiency factors and six meaningfulness factors. The results suggest that the sufficiency factors are more generic, while the meaningfulness factors relate to the individual’s professional drive and occupational focus. Also, it is observed that the two sets of factors seem to be non-sequential, leading us to propose an alternative two-factor matrix that highlights the intricate challenges associated with enacting organizational routines. Based on the new conceptualization, four ways in which staff engage in organizational routines and their respective consequences are outlined and discussed.

## 2. Organizational routines in primary healthcare

According to Feldman and Pentland (2003), organizational routines refer to the patterned behaviors and actions that are collectively enacted by members of an organization. More precisely, the authors define organizational routines as “*repetitive, recognizable patterns of interdependent actions, carried out by multiple actors*” (Feldman and Pentland, 2003, p. 95). These routines are the building blocks of many organizational activities and crucial for the functioning and stability of organizations in that they guide people’s behavior and can function as a mental handrail in operations (Cyert and March, 1963; March and Simon, 1958). As such, organizational routines are an embedded part of most organizations with the consequence that they become ubiquitous in the way tasks are performed (Becker, 2004).

Traditionally, organizational routines have been seen as mindless behaviors (Ashforth and Fried, 1988; Gersick and Hackman, 1990), as they economize on cognitive resources and reduce uncertainty (Becker, 2004). However, during the past decades, research has shifted to acknowledge the performativity of routines (Feldman and Pentland, 2003), emphasizing the agency of individuals and the dual nature conceptualized through a *performative* and an *ostensive* dimension (Pentland and Feldman, 2005). Organizational routines have thus become recognized as mindful and effortful accomplishments (Pentland and Rueter, 1994). As individuals bring their personal agendas to routine performances, personal goals may sometimes not align with the intention of the organizational routine (D'Adderio and Safavi, 2021). Recent studies also show that organization members’ intentions and roles influence how organizational routines are accomplished and the purposes they serve (Rosales, 2020). Additionally, organizational routines are today understood in relation to the *artifacts* – such as routine descriptions, standard operating procedures or written rules – which normatively describe a desired action pattern and thus influence understanding and actions (Pentland and Feldman, 2008). Such normative artifacts are often in everyday speech (incorrectly) equated with the organizational routine itself. Still, standard operating procedures and other artifacts have been shown to help bridge the gap between an organization’s preferred performance and recurrent interaction patterns that make up the (actual) routine (Becker and Zirpoli, 2008).

In healthcare organizations, organizational routines are shown to be essential for several reasons. First, if enacted properly, they create much-needed stability and resilience (Rytterström *et al.*, 2011). With a constant flow of patients in need of various treatments, they help physicians ensure correct diagnosis and care and, ultimately, patient safety (Greenhalgh, 2008). As such, it has been described as an important “structuring device” that also enables organizational learning (Greenhalgh, 2008, p. 1,269). Organizational routines also support continuity and equal care, which can be challenging in a context like primary healthcare, which suffers from increased turnover and a staff shortage. The work environment is also characterized by a high workload, stress and sick leave (Johansson *et al.*, 2019). Routines guide established staff and temporary employees in their daily practice and facilitate the coordination of tasks (Okhuysen and Bechky, 2009; Prætorius, 2016). Additionally, their structuring function, often supported by standard operational procedures, is essential from a workplace safety perspective (Greenhalgh, 2008), as deviations can be devastating and cause injury to patients and staff (Banja, 2010).

However, all these potential benefits for staff and patients can only be “harvested” if practitioners engage in, uphold or enact the organizational routines (Greenhalgh, 2007, 2008), where “enactment” should be understood in line with Weick (1988), who argues that enactment is the effects of people’s actions as they bring structures and events into existence. Enacting an organizational routine thus puts it in existence (Pentland and Feldman, 2005), which can bring both intended and unintended consequences (Fjällström *et al.*, 2022) With organizational routines also being a collective phenomenon (Feldman and Pentland, 2003), a lack of enactment implies that the routine ceases to exist beyond the policy level and, at best, becomes written-down descriptions or individual habits (Reich and Williams, 2003). The subsequent question is, thus, what is needed for organizational routines to be perceived as meaningful and worth upholding despite their negative associations? A first step towards shedding light on this is to explore the notion of meaningfulness and the question of what makes work perceived as meaningful more generally.

## 3. Factors that make work meaningful

While there is little consensus on exactly what factors make employees experience a subjective sense of meaningfulness in their work (Martikainen *et al.*, 2022), many studies have focused on factors at the individual, organizational and societal levels (Lysova *et al.*, 2019). In a recent review by Bailey *et al.* (2019), the authors propose that factors can be grouped into four categories of prerequisites of meaningful work. The authors describe these as “antecedent factors” and categorize them into *job design*, *leadership and management*, *workplace relationships* and *organizational-level factors*. Lysova *et al.* (2019) provide a similar categorization where they propose four levels of factors from individual to societal. While they argue that there is no guarantee that the factors in these groups always lead to perceived meaningfulness in work, various empirical studies have shown that the presence of the factors can contribute to a sense of meaningfulness, and a lack thereof can deteriorate meaningfulness, both individually and in combination with other factors (Lysova *et al.*, 2019).

According to Bailey *et al.* (2019), *job design* is the category that has received the most attention from scholars. Several studies have shown positive associations between job design factors – such as skill variety, task significance and task identity – and meaningful work (Johns *et al., 1992*; Schnell *et al.*, 2013). Work-role fit and specific job characteristics, like challenging and autonomous work, have also been linked to an increased sense of meaningfulness in work (May *et al.*, 2004; Kahn, 1990). As such, this category of factors implies that meaningful work is associated with the level of alignment between personal values and the organization’s purpose or goal, which according to Lysova *et al.* (2019), can be achieved either by adjusting job characteristics or by “job crafting” (i.e. individually driven change processes). In other words, meaningfulness could thus result from the structure and design of organizations and their fit to individual preferences.

Somewhat related to the *job design* is the category *leadership and management* (Bailey *et al.*, 2019). Studies in this category have focused on the role (or type) of leadership and its impact on meaningful work (Lysova *et al.*, 2019). For example, research has shown that transformational leadership, leader sense-giving and support from leaders can be important for individuals’ sense of meaningfulness in their work (Arnold *et al.*, 2007; Carton, 2018; Tummers and Knies, 2013; Duchon and Plowman, 2005; Gloria and Steinhardt, 2016). Moreover, certain management styles that by co-workers are perceived as participative are also shown to contribute to higher levels of perceived meaningfulness (McCrea *et al.*, 2011; Pavlish and Hunt, 2012), which clearly relate to Lysova *et al.'s* (2019, p. 380) statement that *“[…]* *leadership needs to be authentic*.” On the contrary, and not so surprising, is that leadership styles that are non-authentic, divisive or abusive are shown to reduce meaningfulness in work (Pavlish and Hunt, 2012; Bailey and Madden, 2016). Consequently, employees’ sense of meaningful work is dependent not only on the work-role fit but also on the behavior of managers and leaders.

The third category that Bailey *et al.* (2019) identify is categorized under the label *workplace relationships* and relates to the social dynamics of the workplace, which, in the review by Lysova *et al*. (2019), is referred to as the *social context at work*. Unsurprisingly, it is observed that positive workplace relationships are important in fostering meaningful work. Moreover, factors such as having a good work-life balance (Munn, 2013) or experiencing a sense of unity are shown to bring meaningfulness to work (Colbert *et al.*, 2016; Lips-Wiersma and Morris, 2009). This category consequently relates to lateral-level interactions between individual co-workers and co-worker groups.

Finally, in their review, Bailey *et al.* (2019) identify a category that they call *organizational-level factors,* which according to the authors, is a less researched category. However, in the review, the authors highlight that factors such as spiritual or learning-focused work climates are shown to contribute to a sense of meaningful work (Duchon and Plowman, 2005; Pavlish and Hunt, 2012). Lysova *et al*. (2019) also describe this in terms of *organizational culture*, and a study by Schnell *et al.* (2013, p. 543) shows that “*socio-moral climate and organizational self-transcendent orientation contribute positively to the prediction of meaning in work*.” Also, certain characteristics like autonomy, self-selected teams and a community orientation have been linked to meaningful work (Schnell *et al.*, 2013).

All in all, there are several different types of factors, on different levels, that have been shown to contribute to a sense of meaningful work (Bailey *et al.*, 2019; Lysova *et al.*, 2019). For a summary of the four categories and examples of factors involved, see Table 1.

**Table 1:** Summary of antecedent factors for meaningful work

Categories of antecedent factors	Description	Examples of factors
Job design	Factors related to the form and content of employment	*Skill variety, task significance, task identity, job enrichment and autonomous work*
Leadership and management	Factors related to subordinate interaction with management	*Transformational leadership, leader sense-giving, strong leader-member exchange and supervisor support*
Workplace relationships	Factors related to balance and experience of lateral interactions	*Positive workplace relationships, work-life balance and recognition by peers*
Organizational-level factors	Factors related to organizational cultural orientation	*Spiritual or learning-focused work climates, community orientation and self-transcendent orientation*

**Source(s):** Table created by authors

Important to note is that the various factors are not only interrelated and influence each other but are subjective and contextually dependent and thus vary with different jobs and organizational settings (Lysova *et al.*, 2019). Research also points to the fact that the factors that drive meaningfulness are easily eroded and thus have a negative impact (Bailey and Madden, 2016; Lysova *et al.,* 2019). For example, leadership practice can both enable and erode a sense of meaningfulness (Lysova *et al.*, 2019). Thus, a holistic view of what makes work meaningful can differ depending on the type of work tasks, which often can involve aspects perceived as repetitive and tedious (Bailey and Madden, 2016). The factors described in existing research for the meaningfulness of work at large are thus likely to differ from what influences subjective experiences of meaningfulness or meaninglessness of routines.

## 4. Towards a two-factor model of meaningful organizational routines

**Figure 1:**
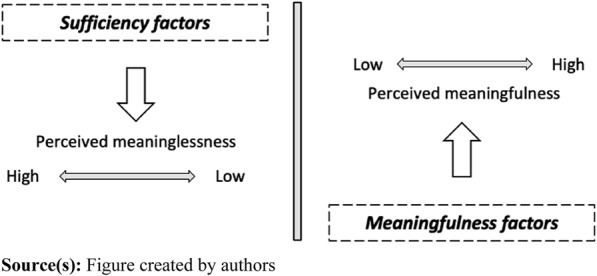
A two-factor model of meaningful organizational routines

Building on the observations by Bailey and Madden (2016) and Lysova *et al.* (2019), the *meaningfulness of organizational routines* might be subject to a dual nature, analogously to the two-factor theory of motivation presented by Herzberg (1964, 2005). In their writings, Bailey and Madden (2016, p. 56) reference Herzberg (1964) and conclude that a different set of factors “*seem to drive a sense of meaninglessness”* than “*those associated with meaningfulness*.” Furthermore, they argue that their research shows that *“[…]* *meaningfulness is largely something that individuals find for themselves in their work, but meaninglessness is something that organizations and leaders can actively cause”* (Bailey and Madden, 2016, p. 58). These observations clearly resemble Herzberg's (1964, 2005) conceptualization regarding the intricate dynamics that shape employee motivation at the workplace. He argued that motivation can be separated into two sets of factors. These two factors influence individuals’ motivation as they either help to *avoid dissatisfaction* or *create satisfaction*. He argued that fulfillment of the former (so-called “*hygiene factors”*) can never lead to satisfaction but solely serve as a way for employees to avoid dissatisfaction. However, the latter (so-called “*motivational factors”*) can. More specifically, *hygiene factors* are described as being external elements related to, for example, the work environment and employment conditions. In contrast, *motivational factors* are “intrinsic” and, as such, inherent to the nature of work and individuals' experiences (Herzberg, 1964, 2005). Also, Lysova *et al.* (2019) indicate that the factors associated with meaningful work have inherent differences, as some have positive and others have negative influences on the perception of meaningfulness. For example, job crafting, in terms of alignment between personal preferences and the content of work, is shown to have a positive impact (Grant, 2007), while a bureaucratic culture has been shown to be able to deteriorate a sense of meaningfulness (Lee *et al.*, 2017). Analogously, a two-factor model of meaningful organizational routines would thus imply two sets of meaningfulness-related factors: we propose a set of *meaningfulness factors* (that can enable meaningfulness) and a set of *sufficiency factors* (that can reduce meaninglessness). Based on these observations, we have deduced a two-factor model for meaningful organizational routines (see Figure 1), where one set of factors merely can eliminate meaningfulness, while another set can enable meaningfulness in routines. In the method section, we will further discuss how these two factors are used as sensitizing concepts in the thematic-based analysis (Bowen, 2006) and as such will be revisited in the discussion section.

## 5. Method

Given the premise of the study and the outlined research question and aim in mind, we adhere to a constructivist ontology and interpretivist epistemology (Cunliffe, 2011). This means that we approach reality as a social construction where our role as researchers is to interpret and make sense of the social phenomena studied (Berger and Luckmann, 1967). In this case, with meaningfulness being subjective and experienced by the individual (Martikainen *et al.*, 2022), in combination with organizational routines being collective and thus socially constructed (Feldman and Pentland, 2003), we need to capture the respondents' understanding of both meaningfulness and meaninglessness of routines and *how* that relates to the individual and the collective. Consequently, conducting qualitative semi-structured interviews with the professional categories involved in various routines was deemed suitable to create such understanding.

### 5.1 Research setting

To understand what characterizes organizational routines as found meaningful or meaningless, respondents were selected based on their occupational roles within a county healthcare organization covering about 30 primary healthcare units (HCUs) and small infirmaries. To gain access to an adequate number of respondents and ensure a wider set of data with similar contextual conditions, respondents were identified and invited from three out of four HCUs within one of the county regions, called *Region North.* The three HCUs included (referred to as *East, West* and *South*) have a broad range of medical professionals and administrative staff, including doctors, district nurses, nurses, midwives, assistant nurses, rehabilitation staff, behavioral scientists, medical secretaries and dieticians. The decision to select respondents from three out of the four units had both practical and methodological rationales. Practically, the fourth unit was located far away from the other three, in a much less densely populated area, and was thus harder to access. Methodologically, this implied that the fourth HCU somewhat differed in its operation and thus is deemed as more of an outlier. The three included units have between 20 and 60 employees and list between 6,000 and 13,500 patients each.

### 5.2 Data collection

In preparation for the interviews, a semi-structured interview guide was developed and pre-tested. The guide mainly focused on examples of organizational routines in the respondents’ daily work that were perceived as meaningful and meaningless, with prodding questions to get further insights into *why* they perceived them as such and *what* this meant for them. To capture the collective nature of routines, involving the interaction of multiple actors in different roles (Becker, 2004), the interviews also included questions on the respondents' respective roles and their involvement with other professionals and interaction in the routines. To address aspects of meaningfulness in general, questions on their choice of career and motivation in their daily work were also included.

As an introduction to the interviews, a presentation to the staff at each HCU was given. At these, the aims of the study and its scope, as well as the concept of organizational routines, were presented. More precisely, we explained *how* organizational routines are conceptualized in research and *how* our interest was not primarily in routine descriptions, standard operating procedures or care pathways. Based on prior experiences, we believed this to be important as routines in the healthcare context often are formalized in such a way (Rytterström *et al.*, 2009) and as people may tend to mistake the written procedures or “artifacts” for the entire routine (Pentland and Feldman, 2008). To capture potential occupational differences in perceptions of the meaningfulness of routines, interviews were undertaken with three different professional categories or roles from each HCU (*managers/administrative staff*, *nurses/other medical staff* and *physicians*). When referring to “roles,” it should be noted that this is not to be interpreted in a sociological sense but rather in its more everyday meaning, referring to formal (professional) involvement and official title in their respective organization. It is likely that the different roles included in this study perceive meaningfulness differently due to the difference in responsibility, workplace relationships and leadership (Bailey *et al.*, 2019).

Each interview started with a reiteration of the study’s aim and focus on organizational routines, and respondents were asked to provide examples of organizational routines they were a part of that they found meaningful and meaningless. They were encouraged to think of organizational routines involving different roles. With our interest being individuals’ subjective experiences of the routines, we have an emic approach and are guided by the respondents’ own understandings of the routines (Pentland and Feldman, 2008). As such, our intention was only to capture their point of view and not the complete routine whose complexity they may be unaware of (Pentland and Feldman, 2008). In this study, this is instrumental in capturing what is significant to respondents and the perspectives of different professional categories.

In total, 24 semi-structured interviews were conducted, corresponding to about 18 h of recorded material. All interviews were transcribed and validated by respondents. Table 2 provides an overview of the respondents, their professional titles, organizational belonging and interview length.

**Table 2:** Overview of interviews and respondents

#	Category	Professional title	HCU	Length of interview (min)
1	Managers and administrative staff	Head of healthcare unit	South	42 min
2		Head of healthcare unit	West	57 min
3		Head of healthcare unit	East	42 min
4		Administrator	South	40 min
5		Medical secretary	West	47 min
6		Medical secretary	East	41 min
7		Medical secretary	East	36 min
8		Administrator	South	45 min
9	Nurses and other medical staff (non-physicians)	Specialist nurse	South	44 min
10		Physiotherapist	South	47 min
11		Physiotherapist	West	58 min
12		Occupational therapist	West	50 min
13		Specialist nurse	East	48 min
14		Specialist nurse	East	49 min
15		Assistant nurse	East	39 min
16		Midwife	East	40 min
17	Physicians	Senior specialist	South	55 min
18		Resident	South	40 min
19		Resident	East	49 min
20		Senior specialist	West	36 min
21		Resident	West	39 min
22		Resident	West	41 min
23		Registered medical practitioner	East	36 min
24		Resident	East	40 min

**Source(s):** Table created by authors

### 5.3 Data analysis

The collected material was analyzed in two different steps, at different points in time. First, because the interviews were conducted independently by two researchers in the research team, several so-called “swift analyses” were undertaken to capture and share the instant impression from the interviews. This provided a joint opportunity to reflect on the insights and make notes about interesting observations. To undertake such “swift analysis” close to conducting the interviews is appropriate when there is an expected time lapse between data collection, transcription and analysis.

The second step was more structured and followed the suggestions from Braun and Clarke (2006) about the process of thematic coding on a latent level. Our decision to use thematic analysis on a latent level is motivated by its capacity to combine flexibility and rigor in the process of “*identifying, analyzing and reporting patterns (themes) within data*” (Braun and Clarke, 2006, p. 79). The structured stepwise approach ensures rigor in the process, moving researchers from familiarizing themselves with the data to writing up the results (Braun and Clarke, 2006). At the same time, the process offers flexibility as it enables us to go beyond the surface-level (semantic) meanings and delve deeper into implicit interpretations (the latent level) of what the respondents find meaningful or meaningless (Boyatzis, 1998; Braun and Clarke, 2006).

To undertake a thematic analysis on a latent level further implies the potential of, in part, using a more template-based strategy (Braun and Clarke, 2006). For us, this implies the opportunity to code based on the above-presented two-factor model of meaningful organizational routines deduced from Bailey and Madden (2016) and Lysova *et al.* (2019) (see Figure 1). The two categories of factors in the model could be seen as sensitizing concepts (Bowen, 2006) in that they enabled us to code the material and search for themes within these two categories but still allowed for inductiveness. Practically, this meant that we coded the material inductively but also tried to distinguish between factors that reduce meaninglessness (the “sufficiency factors”) and those that enable meaningfulness (the “meaningfulness factors”). Aligned with the suggested approach by Braun and Clarke (2006), coding was done systematically across all interviews, accumulating data relevant to each code. In the analysis of the respondents' descriptions of routines perceived as meaningful or meaningless, we particularly looked for why the routine was perceived as such if there were any characteristics of the routine or what it did.

After that, the iterative process of collating codes into potential themes (in the results called “factors”) took place. At this stage, while one of the co-authors took the lead in the coding process, in line with Barbour (2001), concurrent discussions between the two co-authors were held, jointly looking over the coding of interview segments and emergent themes to ensure the trustworthiness of our interpretations (Nowell *et al.*, 2017). In total, nine *sufficiency factors* and six *meaningfulness factors* were identified. Thereafter, the development of clear definitions and descriptive names for each factor took place, followed by identifying representative extract examples and quotes. For an overview of factors and definitions, see Table 3. Additionally, we looked for descriptions of what the perception of meaningfulness or meaninglessness meant for respondents and how it influenced them and their enactment of the routines. Finally, the results from the thematic analysis were contrasted with previously identified factors and juxtaposed with the outlined two-factor model.

**Table 3:** Summary of meaningfulness and sufficiency factors

Professional roles	Sufficiency factors	Description of factors	Meaningfulness factors	Description of factors
Managers and administrators	Facilitating work	*Simple and easy to follow. Efficient, time saving*	Locally anchored	*Updated and practical in the context. Including all involved working with it or impacted*
	Enabling work (managers)	*Necessary for doing the work. Having time and resources*	Supporting correct care	*Helping the patient. Help doing things right when insecure*
	Contributing	*Understanding why something is done, purpose. Seeing that it leads to something – use*		
Nurses and other medical staff	Efficient and timesaving	*Efficient, time saving. Facilitating work*	Making use of their competence	*Within their power. Making use of their expertise*
	Having an effect	*Filling clear function. Visible results*	Benefiting the patient	*Having a positive impact on the patient. Assuring good and equal treatment*
	Anchored and prioritized	*Taking all involved into account. Prioritized by physicians and management*		
Physicians	Using competencies efficiently	*Not wasting time and resources. Efficient for patients*	Assuring quality of care	*Scientifically based – in line with national procedures. Saving lives and preventing mistakes*
	In line with personal values	*Contributing to the patient.* *Not imposed for others’ purposes*	Facilitating collaboration and shared responsibility	*Enabling cooperation and learning. Making care a joint concern*
	Locally anchored	*Adaptable to circumstances* *Grounded and applicable in local operations*		

**Source(s):** Table created by authors

## 6. Results

The first part of the results is presented consecutively, by role category and oriented towards the research question posed in the introduction: *What characterizes organizational routines that are perceived as meaningful?* To do so, for each role category, we begin by providing the respondents' view of their roles and work tasks and how they perceive routines in their work in general. After that, we will present the identified characterizing factors.

### 6.1 Managers and administrators

The first category of respondents consists of staff working as medical secretaries, heads of healthcare units and general administrators. Respondents in this category all describe their function in terms of helping people, including both patients and their colleagues. As described by one respondent, “*[…]* *Our whole profession is about making things easier for others. So, we make it easier for doctors to write medical records, for example, and […] so that everything goes smoothly, and they can concentrate on seeing patients instead”* (#6 Medical secretary). Respondents highlight that they have an important role working in healthcare. Although they are not working directly with treating patients, they find that meeting patients and helping them is motivational. The managers all see the staff as their key focus. Being there and supporting them and their development and well-being is essential.

Regarding organizational routines, respondents perceive them as essential tools facilitating everyday work, helping with structure and knowing what to do or something to fall back on even when people may be stressed and tired. As such, the routines constitute a critical aspect of the work environment, staff well-being and patient safety. In addition, and particularly for the administrative staff, routines are seen as providing a sense of security in doing the right thing, as they lack medical training. Some of the routines described by the managers and administrators include the introduction of new staff, the follow-up on canceled appointments and the handling of patients’ charts.

#### 6.1.1 Sufficiency factors

For organizational routines not to be considered meaningless, it is found necessary by the administrative staff that the routine makes their work easier and more efficient rather than the opposite. The routine itself cannot be perceived as too complex. In essence, it is a matter of routines having the potential to *facilitate work.* The administrative staff highlights that they need to understand *why* a routine exists and see that it has a concrete use and leads to something relevant. As put by a respondent, it becomes meaningless when you do not get anywhere with the work effort you put in: *“[…]* *this thing where you have to sit and do the same thing several, several times and nothing happens”* (#5 Medical secretary). This can be summarized as a factor of the routine *contributing.* The managers highlight that for a routine not to be perceived as meaningless, it must be essential for work to be done, and there must be sufficient time set aside to uphold it. Consequently, for them, the routine should have a function of *enabling work.*

#### 6.1.2 Meaningfulness factors

For routines to be considered meaningful by administrative staff, it is essential that the routine practice include all of those who are (or should be) involved, that those individuals are committed and that everyone is a part of it and goes along with it. There must be an understanding that more professions are affected by the routine and that no one feels overrun. Furthermore, for the routine to be perceived as meaningful, it also needs to be updated, work in the context and everyone should feel included. In other words, the routine must be *locally anchored*. Another factor contributing to the meaningfulness of routines for administrative staff is that it is perceived as *supporting patient care*. As not all of those who hold administrative roles have medical training, the routine can be a way for them to ensure that patients are treated well. As described by one of the respondents: *“I think it becomes meaningful when I feel that I have use of the routine as a support”* (# 4 Administrator). It is seen as meaningful when the routine can relieve uncertainty and insecurity and when it contributes to helping the patients and that patients are taken care of the way they would like to be met when in contact with primary care.

### 6.2 Nurses and other medical staff

This second category of respondents consisted of, for example, specialist nurses, physiotherapists, midwives and assistant nurses. Similar to what managers and administrators responded, the nurses and other medical staff also stress that their job is about helping people and making a difference. Meeting the patients is often mentioned as the best part of their workday, and seeing a broader picture of their lives, working preventative and health-promoting rather than only fixing an illness, is vital. They appreciate having some control over their work, highlight the variation in their job and see it as developing to solve problems and learn new things.

From a general point of view, they see routines as a support to work independently and do the right things. As expressed by a respondent: *“[…]* *that someone has thought about and […] for the large mass of a patient group, how is it smartest to solve it so that we all do the same”* (#14 Specialist nurse). As such, the routines assure patient safety as nothing gets missed or forgotten. Also, it enables nurses and other medical staff to guide patients, knowing where their patients are going next. It is seen as necessary that everyone does things in the same way so that patients get equal treatment, but at the same time, respondents stress that they must be responsive and cannot be too rigid. For respondents in this category, routines are not only about treatment but also the way they have decided to organize work in the HCU. Routines are thus seen as a way of structuring things, facilitating the workflow and making it efficient and smooth both for staff and the patient. Routines chosen as examples in the interviews include handling patient-initiated contacts, following up on patients with chronic diseases such as diabetes and investigations of conditions such as dementia, asthma or hearing loss.

#### 6.2.1 Sufficiency factors

For nurses and other medical staff, the themes of *efficiency and timesaving* are very prominent when describing what is needed for a routine not to be perceived as meaningless. They describe that the routines need to make the work easier and reduce unnecessary or redundant extra work and wasted time. With stress, lack of time and resources, they want to reduce the time and effort spent: “*If you don’t have the time, then you do it as quickly as possible. Then maybe you don’t go and check what our routine is or what we are supposed to do*” (#11 Physiotherapist). Consequently, routines must facilitate their work and increase predictability and having to think about the next steps. For routines not to be considered meaningless by the respondents, it is also crucial that the routine has a clear function and that results are visible. It is expressed that it is important for them to understand the whole chain of steps and that what has been done can be followed up. For the routine to *have an effect* is thus another important sufficiency factor for this category. Routines should also be anchored among all the categories of roles that are involved, thought through and considering how it affects each of them. If the routine is externally imposed, doesn’t work for everyone and it is not continually communicated about why it is as it is, it will fail. Consequently, the routines need to be *anchored and prioritized*. If this group sees that other staff, such as physicians, do not prioritize the routine and do things in a way that suits them better or that the routine is not prioritized by management, the meaningfulness is eroded, and the routine is not upheld.

#### 6.2.2 Meaningfulness factors

On the contrary, for routines to be considered meaningful by nurses and other medical staff, their role in it needs to be recognized as valuable, and they need to feel that they have the mandate to perform their part of the routine independently. A key factor for them to perceive a routine as meaningful is that it is *making use of their competence*; when they are capable and trusted to perform an element of care, it is within their control, and they own their part in it. Routines are also perceived as meaningful if they are perceived as *benefiting the patient*, for example, that they contribute to the patient’s well-being and quality of life, even before becoming ill, that they ensure quick and good treatment and that all patients are treated equally. It is seen as meaningful when the routine contributes to being able to help the patient and that following it has an impact on them.

### 6.3 Physicians

The third and final category, physicians, includes all levels of physicians, from residents to registered medical practitioners to senior specialists. What they have in common, however, is that they are Medical Doctors (MDs) and have completed their formal education. The physicians' descriptions of their work and motivations, quite naturally, center on the patients, *“doing something good”* and making a difference. They also describe meeting with other humans, following unique lives and being of importance to other people as key to their role and motivation. On the other hand, they also describe the intellectual challenges, the extensive knowledge needed, how the role requires constant development and that they must make crucial decisions independently. It is described as a multifaceted role but rewarding because they get to work with something they have long strived and studied for.

The physicians see routines as a support in knowing that they are “doing the right thing” and that everyone does things in the same way. For them, routines are agreed-upon ways of working so that everyone knows what is expected of them and what should be done when. Routines are perceived to create a sense of security and reduce the risk of making errors or harming the patient. One respondent is more critical of the importance of routines and says: “*[…]* *what you yourself, and probably others, think that a doctor works with, what you think when you start, is probably not what reality looks like for many. Rather […] the purely medical becomes less and less today, [upholding] the routines becomes* *the main purpose*” (#21 Resident). In other words, this respondent thinks the emphasis on routines and procedures comes from a fear of doing the wrong thing, which means that medical care becomes less dependent on the individuals’ knowledge. While physicians often focused on care pathways and treatment routines, for example, about how to handle patients with urinary tract infections or high blood pressure, other examples of organizational routines they brought up include regular internal consultation sessions in the health center and the process for patient-initiated contacts online.

#### 6.3.1 Sufficiency factors

Like the other professional groups interviewed, the physicians talk about the importance of routines being efficient and timesaving. For the physicians, it is, however, key that the routine should not lead to additional work and that the valuable resource that the physician’s time constitutes should not be wasted. The aim of timesaving is to be able to help more patients. For routines not to be considered meaningless by physicians, they must enable good use of resources and competencies, i.e. *using competencies efficiently.* Furthermore, for the physicians, for a routine not to be considered meaningless, it needs to contribute to the patient. As such, it is important to them that the routine is *in line with their values* of patient care and not based on “externally imposed demands” based on other premises, such as documentation, administration or funding. The physicians also highlight that the routines do not risk becoming meaningless if they are founded on local needs, shaped according to local circumstances and valuable to those performing them. Also, they want routines to be simple enough for them to be able to adapt them to the patient’s cases. We summarized this as a factor of the routine being *locally anchored.*

#### 6.3.2 Meaningfulness factors

For physicians to perceive a routine as meaningful, it is, however, important to them that it *assures quality of care*, which entails it being research-based and in line with national regulations and procedures. As put by one respondent: “*Because they aim to identify* *and* *catch potentially life-threatening disease at a stage where you can do something about it [laughter], so to speak. And there, I think it feels like a routine that is also anchored in the entire care chain to really find and cure people from cancer*” (#19 Resident). As such, the routines contribute to saving lives, preventing mistakes and ensuring that something is not missed, thus providing a sense of security. Another factor for meaningfulness for the physicians is that the routine *facilitates collaboration and shared responsibility.* They highlight that it is possible to be alone in their role and routines that give opportunities for shared reflection, making the care a joint concern, are meaningful to them. As one of our respondents explains it, “*[…]* *you feel that you are in a context, you feel that you are working together”* (#22 Resident). Meaningful routines can thus allow them to learn from each other and give the patient the best conditions.

### 6.4 An aggregated view of the roles and influencing factors

As outlined above, there are, in total, nine *sufficiency factors* identified, three for each category. Additionally, six *meaningfulness factors* have been identified. In the following table, we summarize the identified factors and their characteristics for the three professional categories (see Table 3).

## 7. Discussion

In line with Bailey and Madden’s (2016) observation that there is a difference between driving and eroding factors or meaningfulness, we have identified six meaningfulness factors and nine sufficiency factors. The identification of these factors answers the first part of our research question in terms of *what characterizes organizational routines that are perceived as meaningful*. With these factors in mind, we will start our discussion by reflecting on the similarities and differences between the three professional groups and how the factors relate to those previously identified.

Similarities can clearly be identified among the various professional groups. For example, for both *managers and administrators* and *nurses and other medical staff*, the notion of “making things more efficient” is significant. Also, among physicians, this theme is present. However, they express this more in relation to their own role, in terms of not wasting “physician resources.” This is very much in line with the observations made by Andersson *et al.* (2022) when it comes to physicians finding their time to be a highly valuable resource. In the mentioned study, it was shown how physicians experienced managerial work as less meaningful and important than clinical work. Another similarity is that all the professional categories highlight that for a routine not to be considered meaningless, it must have some of the elements considered “inherent in routines.” In other words, it is in various ways stressed that routines should be simple and easy to follow, facilitate work and have a clear purpose. In the context of primary care, with limited time and resources, there also needs to be sufficient time and resources to be able to follow them (*cf.* Rytterström *et al.*, 2011). Although the *sufficiency factors* are quite similar in nature, there are differences between what aspects are highlighted by the different roles, stemming from the perspective of the position and function of the professional role. Among the *meaningfulness factors*, the differences become more apparent. For example, all categories see the benefit for the patient as contributing to meaningfulness, but from very different perspectives. While “quality of care” is a strong meaningfulness factor, and the routine contributing to the patient is necessary in order not to be meaningless for physicians, for the group *managers and administrators,* the sense of meaningfulness lies in the routine supporting this care. Thereby, as suggested by Bailey and Madden (2016), they find meaningfulness in crafting a broader importance of their role. Variations of the theme of the routine being anchored are present for all the groups; however, for *managers and administrators,* this is more about inclusion, while for *the physicians,* this refers more to adaptability.

Rather unsurprisingly, some of the meaningfulness factors identified also overlap with what is found in previous research regarding what makes work meaningful. For example, the *facilitating collaboration and shared responsibility* factor, which is essential for *physicians* to perceive a routine as meaningful, clearly resembles some of the factors identified in what Bailey *et al.* (2019) conceptualize as *workplace relationships* and by Lysova *et al.* (2019) as *social context at work.* Physicians highlight the importance of meaningful routines enabling collaboration among peers, like what Colbert *et al.* (2016) identify in their study, which shows how collective support improves meaningfulness in work. Although this connection is a less clear factor for the other categories, the importance of workplace relationships comes across as an underlying value, although expressed differently. The factor “local anchoring” for *managers and administrators* is about inclusion and no group being overrun, and for *nurses and other medical staff,* it means making use of their competence and being respected by their colleagues. Moreover, among the identified factors, some examples could be explained in terms of *job design* (Bailey *et al.*, 2019), in particular “task importance,” which is reflected in the importance of the routine having a purpose, having an effect and making a positive impact.

Consequently, the meaningfulness of routines is somewhat linked to aspects identified as contributing to the perceived meaningfulness of work (Bailey *et al.*, 2019; Lysova *et al.*, 2019), which is inherently subjective and individual. However, organizational routines are, by definition, collective in their nature, in the sense that they are reliant on shared efforts to be enacted and the benefits thereof to be realized (Feldman and Pentland, 2003). Meaningful routines thus become an individual-collective dichotomy. This observation very much aligns with the prevalent conceptualizations of routines in terms of the ostensive-performative dimensions, where individual actors, through their enactment, can drive change in routines, while the collective nature creates stability (Feldman and Pentland, 2003; Becker, 2004). As such, it supports the idea that (meaningful) organizational routines are “effortful accomplishments” (Pentland and Rueter, 1994), where a tension might occur between individual (subjective) sense of meaningfulness and the collective enactment. As shown, what is perceived as meaningful for one professional group might not be the same as for others. Also, the *sufficiency factors* and the *meaningfulness factors* of routines seem to be driven by two different logics or be of different natures.

On the one hand, the sufficiency factors identified seem to be more generic in the sense that they are related to the overall understanding of *why a routine exists* and *how it can contribute*. Still, they seem to go beyond routines merely being non-useful in that it has to do with anchoring, prioritizations and the need for interrelatedness with others. On the other hand, the meaningfulness factors seem to be *associated with the professional raison d'être* – in other words, the professional drive and occupational focus of various groups. For example, physicians who identify with having the patient’s best interest in mind seem to find meaningfulness in those routines that support and uphold patients’ safety and the quality of care, while administrative staff who identify and are passionate about more functionalist aspects of the organization find meaningfulness in routines that ensure efficiency in their workflow. This is also in line with previous observations regarding the benefits of alignment between the personal motives of healthcare professionals and organizational goals (Kjellström *et al.*, 2017).

**Figure 2:**
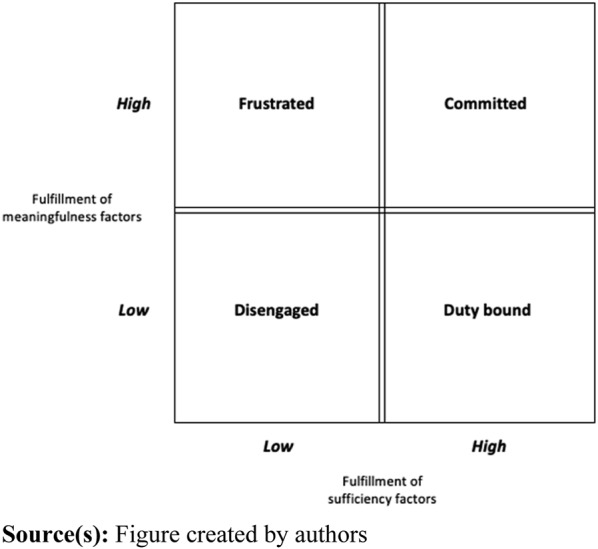
Organizational routine enactment based on influencing factors

Consequently, our findings thus give some support to the notion of a two-factor model being applicable (Herzberg, 1964, 2005; Bailey and Madden, 2016) but are at odds with the claims that *“… meaningfulness is largely something that individuals find for themselves in their work, but meaninglessness is something that organizations and leaders can actively cause”* (Bailey and Madden, 2016, p. 58). The conflicting observations seem to indicate that these factors are not necessarily sequential or mutually exclusive when it comes to organizational routines. While an organizational routine may be frustrating and perceived as meaningless in some respects, due to its collective nature, there may still be factors of meaningfulness still present (Feldman and Pentland, 2003). Consequently, we argue that for organizational routines, the two types of factors do not build on each other in a two-step fashion, in a way that the sufficiency factors need not be fulfilled before staff are able to find meaningfulness in organizational routines (see Figure 1). For example, as the results indicate, a nurse can find that an organizational routine clearly benefits the patients (*a meaningfulness factor*) but still lacks anchoring or prioritization amongst other staff (*a sufficiency factor*). Likewise, a physician can find that an organizational routine clearly assures the quality of care and is scientifically based (*a meaningfulness factor*) but still lacks local anchoring at the HCU (*a sufficiency factor*). In both cases, the two types of factors are not mutually exclusive. Therefore, we propose a two-factor matrix where *meaningfulness* and *sufficiency factors* can be simultaneously present, and the experience thereof can come at different degrees (see Figure 2).

As illustrated in Figure 2, depending on the level of fulfillment of the two types of factors, a two-factor matrix thus gives rise to four different types of engagement with the organizational routine, which also has implications for whether an organizational routine is upheld as intended or not. In situations with a low level of fulfillment of both meaningfulness and sufficiency factors, professionals would be *disengaged* from the organizational routine. This is prospectively the least beneficial situation because staff, one or several, find the routine completely meaningless and deviations are thus likely, with proven negative consequences thereof (Banja, 2010). In situations where staff experience a high fulfillment of sufficiency factors but a low level of meaningfulness factors, their engagement is *duty bound*. This implies that the routine is upheld, but it’s done out of obligation to other co-workers and not because staff find direct meaning in the organizational routine. Such a situation is less problematic, but still not satisfactory. Alternatively, in a situation where staff finds high meaningfulness in the organizational routine, but no sufficiency factors are present, *frustration* is likely to occur. Respondents explained that the routine is upheld because it provides meaningfulness to the individual. Still, frustration occurs because competencies are, for example, not being used efficiently or there is a sense that the routine is not prioritized by others. Only in situations with high fulfillment of both meaningfulness and sufficiency factors, among all staff involved in a routine, will they be fully committed to enacting and upholding the routine. Consequently, because the enactment of organizational routines is a collective endeavor (Becker, 2004), for routines to be performed without some staff being frustrated, both meaningfulness and sufficiency factors must be found by all involved actors, which becomes the central challenge from an organizational or management point of view. As our findings have shown, what enables meaningfulness in routines for one profession might be what erodes meaningfulness for another. Consequently, identification of misalignment among various staff categories involved in a routine is a good first step towards the creation of meaningful organizational routines.

## 8. Concluding remarks

This paper advances research on meaningful work in primary healthcare by focusing on *the role of meaningfulness in the enactment of organizational routines* rather than considering routine enactment (Feldman and Pentland, 2003; Becker and Zirpoli, 2008) or the meaningfulness of work (Bailey and Madden, 2016; Bailey *et al.*, 2019) separately. A total of nine sufficiency factors and six meaningfulness factors have been identified that explain what *characterizes organizational routines that are perceived as meaningful* among different professional groups in primary healthcare, consequently answering the first part of our research question. Our study has shown that factors that contribute to the experience of routines as not being meaningless are neither sufficient in themselves nor are they always interrelated to the factors making routine perceived as meaningful. Thus, the idea of a sequential two-factor model Bailey and Madden (2016), is put into question. Consequently, in addition to the 15 factors identified and the observation that factors vary among different roles, the developed two-factor matrix is a key contribution illustrating four different ways in which organizational routines are enacted and the consequences thereof. As such, the two-factor matrix takes previous understandings (Bailey and Madden, 2016; Lysova *et al.*, 2019) one step further and answers the second part of our research question in that it explains *how the perception of meaningfulness influences routine enactments.* Our study consequently contributes to the understanding of how different professional roles in the workplace differ in their perceptions of what is meaningful and what erodes meaningfulness. As the various professional roles need to interact in the enactment/performance of organizational routines, their experiences are interlinked. It can thus be concluded that although meaningfulness in work is an individual experience, meaningfulness becomes a relational concept when it comes to organizational routines. As shown in the results, this might lead to *frustration* or that organizational routines are merely upheld due to commitment to others, described as *duty bound*.

By focusing on organizational routines specifically and not work in general, the organizational benefits (and, in this case, also patient safety benefits) stemming from the routines being performed as intended are put in the spotlight. By reducing or eliminating factors that would erode meaningfulness for some co-workers, organizational routines can be better upheld. While many of these factors stem from contextual conditions in healthcare, such as lack of time and resources, there are common denominators that can be focused on to ensure that there is a match between the perception of an organizational routine among involved staff and a shared understanding of its purpose and consequences on those involved. All professional roles included in the study highlighted the importance of jointly developing and anchoring the routine so that it works for all involved. The findings thus have implications to enable improved routine practices, which in turn can improve individuals’ psychological work environment, as well as increase resilience to stress, thereby reducing sick leave and turnover, which are important challenges within healthcare.

The above-mentioned call for joint development of organizational routines has both practical and theoretical implications. Theoretically, this implies that future research could examine “job crafting practices” (see. e.g. Wrzesniewski and Dutton, 2001; Zhang and Parker, 2019) applied to organizational routines, which has previously only been explored related to work in general. Practically, managers and staff who experience organizational routines that cause frustration or are enacted solely due to obligations (see Figure 2) should be encouraged to engage in job crafting practices related to those routines, i.e. altering the organizational routine to either align with personal preferences of meaningfulness or fulfill sufficiency factors. However, we argue that engaging in such “routine crafting” should be done with caution due to the role of organizational routines as “structuring devices” in the healthcare setting (Greenhalgh, 2008). As such, all crafting must be done with a good understanding of what can and cannot be done from a patient safety perspective. In other words, merely because an organizational routine can be made more meaningful does not imply it should be changed.
